# The Effect of Low-Level Laser Irradiation on Sperm Motility, and Integrity of the Plasma Membrane and Acrosome in Cryopreserved Bovine Sperm

**DOI:** 10.1371/journal.pone.0121487

**Published:** 2015-03-17

**Authors:** Guilherme Henrique C. Fernandes, Paulo de Tarso Camillo de Carvalho, Andrey Jorge Serra, André Maciel Crespilho, Jean Pierre Schatzman Peron, Cristiano Rossato, Ernesto Cesar Pinto Leal-Junior, Regiane Albertini

**Affiliations:** 1 Postgraduate Program in Rehabilitation Sciences, Universidade Nove de Julho (UNINOVE), São Paulo, SP, Brazil; 2 Postgraduate Program in Biophotonics, Universidade Nove de Julho (UNINOVE), São Paulo, SP, Brazil; 3 Postgraduate Program in Veterinary Medicine—Universidade de Santo Amaro (UNISA) São Paulo, São Paulo, SP, Brazil; 4 Instituto de Ciências Biomédicas da Universidade de São Paulo—USP—São Paulo, São Paulo, SP, Brazil; ENEA, ITALY

## Abstract

**Background and Objective:**

Freezing changes sperm integrity remarkably. Cryopreservation involves cooling, freezing, and thawing and all these contribute to structural damage in sperm, resulting in reduced fertility potential. Low-level laser irradiation (LLLI) could increase energy supply to the cell and cause reactive oxygen species reduction (ROS), contributing to the restoration of oxygen consumption and adenosine triphosphate synthesis (ATP) in the mitochondria. Our goal was to analyze the effects of low-level laser irradiation on sperm motility and integrity of the plasma membrane and acrosome in cryopreserved bovine sperm.

**Study Design/Materials and Methods:**

We analyzed 09 samples of bull semen (*Bos taurus indicus*), divided into three groups: a control group without laser irradiation, a 4J group subjected to a laser irradiation dose of 4 joules, and a 6J group subjected to dose of 6 joules. Samples were divided for the analysis of cell viability and acrosomal membrane integrity using flow cytometry; another portion was used for motion analysis. Irradiation was performed in petri dishes of 30 mm containing 3 ml of semen by an aluminum gallium indium phosphide laser diode with a wavelength of 660 nm, 30 mW power, and energy of 4 and 6 joules for 80 and 120 seconds respectively. Subsequently, the irradiated and control semen samples were subjected to cryopreservation and analyzed by flow cytometry (7AAD and FITC-PSA) using the ISAS - Integrated Semen Analysis System.

**Results:**

Flow cytometry showed an increase in the percentage of live sperm cells and acrosome integrity in relation to control cells when subjected to irradiation of low-power laser in two different doses of 4 and 6 joules (*p* < 0.05). In the analysis of straightness, percentage of cell movement, and motility, a dose of 4 joules was more effective (*p* < 0.05).

**Conclusion:**

We conclude that LLLI may exert beneficial effects in the preservation of live sperm. A dose of 4 joules prior to cryopreservation was more effective than a dose of 6 joules in preserving sperm motility.

## Introduction

Techniques in animal reproduction, such as artificial insemination (AI), are constantly used to increase the quality and quantity of genetic and phenotypically superior calves [1], [2]. The Artificial Insemination in Fixed Time (TAI) allows cows to be inseminated without determining whether they are in heat, because the technique itself induces ovulation. The TAI is widely used to improve genetic quality and herd production volume. The efficiency of the technique is dependent on the semen quality fertilizer and pre-frozen state for post-thaw, which lead to adequate semen motility, vigor, and high viability [3], [4], [5] [6].

Sperm integrity shows remarkable changes with freezing. The cooling, freezing, and thawing involved in cryopreservation all contribute to structural damage and reduced function in sperm, resulting in reduced fertility potential [6], [7], [8].

The cryopreservation process induces morphological changes in semen and damages the plasma membrane, acrosome, and mitochondria. These changes are sufficient to adversely affect the fertilizing capacity of the semen and they significantly accentuate the production of adenosine triphosphate (ATP) and lead to cell death [9]. The sperm plasma membrane regulates the intracellular calcium concentration, particularly the calcium pump by ATP-dependent sodium/calcium and the voltage-dependent calcium channel. [10]. Intracellular calcium movements play a vital role in cell proliferation and in mammalian spermatozoa have a pivotal role in control of sperm motility and acrosome reaction. [11]

Cryopreservation of bull semen adversely affects the sperm membrane integrity, thereby reducing the ability to fertilize the processed sample [12]. In this perspective, effective techniques are needed to protect sperm from adverse effects of cryopreservation.

The low- power laser irradiation of spermatozoa can increase sperm motility as well as velocity can be improved by He-Ne laser irradiation. The first published studies dating back to the year 1984. [13] According to Huang et al. [14], the first Law of Photochemistry says that light photons are absorbed by photoreceptors or chromophores. The low-level laser mechanism at the cellular level has been attributed to the absorption of monochromatic visible radiation and near infrared (NIR) radiation by the cell respiratory chain components. The low-level laser has become an alternative to modulate various biological processes. Depending on the wavelength, dosage, and condition of the irradiated tissue, the laser can induce an anti-inflammatory effect, reducing pain, and accelerating cell proliferation [15].

The biological mechanisms of interaction of the low-power laser aren’t totally known, however, it is known that different kinds of cells don’t behave at same way when irradiated by the same wavelength. For this reason, it is difficult to extrapolate the effects from one cell type to another, but it can be said that at the molecular level, the activation of certain receptors and messengers determine universal biological responses [16].

Therefore, the present study aimed to investigate the effects of low-level laser irradiation on sperm motility, and integrity of the plasma membrane and acrosome in cryopreserved bovine sperm.

## Methods and Materials

All semen handling procedures were performed in accordance with standards established by the Brazilian College of Animal Reproduction (CBRA). The experimental procedures were approved by the Research Ethics Committee of the Universidade Nove de Julho- UNINOVE n.0047/2014 and are in accordance with current legislation.

### Animals

We used 09 semen samples from Nelore bulls (*Bos taurus indicus*), with ages ranging from 24 to 50 months, from the Central Artificial Insemination TAIRANA, (Rod Raposo Tavares, 563 KM.—Presidente Prudente, SP—Brazil). The animals were fed all year round with mineral supplementation and balanced feed and water ad libitum.

### Collection and processing of semen samples

The semen collection was performed according to the health and safety criteria established by the Brazilian College of Animal Reproduction (CBRA). After collection, the semen was taken immediately to the laboratory for sample preparation and physical and morphological analysis. Semen samples were stored in formalin-saline for evaluation of concentration and sperm morphology. The samples of semen used were those at the 50^th^ percentile and above in motility, sperm concentration greater than 200 × 10^6^/mL, and ejaculate volume greater than 5.0 ml.

### Evaluation of sperm concentration

Sperm concentration was determined using Neubarhemocytometer. Samples of semen were diluted with BotuBOV (Botupharma, Botucatu SP—Brazil), until obtaining a final concentration 25 × 10^6^ sperm / mL. The number of spermatozoa was counted in 10 squares with the help of manual counter grid was located with 200X magnification under a phase contrast microscope.

### Experimental design

Viable semen samples obtained from 9 animals were divided into 3 groups: a control group without LLLI (n = 18 Straws); a 4J group treated with LLLI with 4 joules (n = 18 Straws); and a 6J group, exposed to LLLI with 6 joules (n = 18 Straws). The resulting samples were divided into 27 samples to examine cell viability and acrosomal membrane integrity and 27 samples for motion analysis.

### Laser irradiation

The laser used was the Aluminum gallium indium phosphide (AlGaInP) DMC brand, model Photon Laser III (DMC, São Carlos, SP—Brazil) with a wavelength of 660 nm, and variable power from 30 to 100 mW. For this study the equipment was programmed for a power of 30 mW, beam area 0.028 cm and 4 joules of energy for the 4J group (133 seconds of irradiation), and 6 joules for the 6J group (200 seconds of irradiation). Since we performed irradiations directly to petri dishes, we decided to use lower power output of laser device we had in laboratory. The irradiation procedure was carried out in petri dishes of with a total area of 23.6 cm^2^, containing 3 mL of semen with the laser probe was positioned perpendicularly to the plate, within 1 cm, so that the laser beam would illuminate the entire area of the plate without spreading to the outside area, resulting in a power density of 0.0012 W / cm^2^. We choose to perform the irradiation before freezing of samples in order to protect samples of freezing procedures. In order to ensure a uniform procedure was made a protective Ethylene Vinyl Acetate (EVA) black, involving the entire outer area of the plate. The measurement of delivered energy was performed using the Newport multifunction optical meter (Model 1835C, Newport Corporation, Irvine, CA, USA). The samples of control group were not exposed to irradiation, but were exposed to same experimental conditions of all other groups, including the time before freezing procedure start to be performed.

### Cryopreservation

The samples were packaged in French 0.5-mL straws (medium), previously identified with the animal number, the group to which it belongs and collection date. Filling and sealing of the straws was done by an automated system. The semen was cryopreserved (196°C negative) using the Digitcool IMV (IMV—L'Aigle, France). The straws were removed from the machine and immersed in liquid nitrogen, placed in identified according raquis with the experimental group and stored in cryogenic cylinders.

### Analysis of sperm acrosomal integrity and living cells by flow cytometry

The samples were thawed in a water bath at 37°C for 30 seconds and semen placed in a microfuge preheated to 37°C. A 0.5-mL aliquot was removed from each treatment and added to 1.5 mL of 1× PBS solution. An aliquot of 300 μL was withdrawn from the first solution and added to 1.0 mL then centrifuged at 300 g for 10 minutes (Model Mini Spin Minicentrífuga Plus, Eppendorf) in microtubes. The supernatant was discarded and the pellet resuspended in 240 μL based on the second solution and 80 μL resuspended in PBS. Thus, the samples showed a concentration of 25 × 10^6^ sperm/mL. Then 2 μL of 7-amino actinomycin (7AAD) was added, associated with 2 μL of fluorescein isothiocyanate (FITC) + Pisium Sativum Agglutinin (PSA) in the 80 μL sample, and incubated for 8 minutes at room temperature and protected from light. [17], [18]

The analysis of acrosome integrity and live sperm cells were analyzed by flow cytometry that was performed using an Accuri C6 flow cytometer (Accuri Cytometers, Inc. Ann Arbor, MI USA), equipped with a blue and a red laser, two scatter detectors, and four fluorescence detectors (FL1 533/30 nm; FL2 585/40 nm; FL3>670 nm and FL4 675/25 nm) whose range displayed data across 6.2 logs. For this we use fluorescein isothiocyanate (FITC) + Pisium Sativum Agglutinin (PSA) fluorescence was detected at 515–545 nm Fluorescence detector 1 (Fl 1) and 7-amino actinomycin (7AAD) fluorescence was detected at 640 and 680 nm Fluorescence detector 3 (Fl 3). The data analyzed using the Accuri software (CFlow Plus, Ver. 1.0.202.1). The forward scatter and side scatter were plotted, as well as florescence detected by plotting detection on FL-1 versus FL-3. Gating and fluorescence compensation values were set after data collection. A total of 10000 events were analyzed for each sample.

### Sperm motility analysis

Sperm motility was evaluated with the ISAS—Integrated Semen Analysis System. The samples were thawed in a water bath at 37°C for 30 seconds, and 2 μL of the sample were placed in the previously heated reading chamber. Images were captured by a camera attached to a microscope connected to a computer and then analyzed in real time by the software. For this, each sperm cell was identified and its trajectory reconstructed. Parameters analyzed were fast cells; total motility (MT-%); progressive motility (MPRO-%); path velocity (VAP—microns / s) defined by the total distance of the path of each cell divided by the time elapsed; progressive velocity (VSL—microns / s), which is the distance traveled between the beginning and end of the path divided by the elapsed time; curvilinear velocity (VCL—microns / s); the lateral displacement of the head (ALH—microns), the average width of the head and the oscillation of its movement; beat frequency (BCF—Hz), the frequency at which the sperm crosses a path in each direction; straightness (STR-%), measuring the straight path of the sperm cell, is the ratio of VSL / VAP; and linearity (LIN-%), corresponding to the direction of travel, is the ratio of VSL / VCL. [19], [20]

### Statistical Analysis

The Kolmogorov specification test was used to verify the normal statistical distributions and all data were expressed with means ± standard deviation. One-way ANOVA followed by the Newman-Keuls post-hoc test were used for the comparisons with GraphPad Prism software (version 5.0, GraphPad Software, Inc., La Jolla, CA, USA). A *p* values of p ≤ 0.05 was considered significant.

## Results

### Sperm viability and acrosome membrane integrity determined by flow cytometry

**Fig 1:**
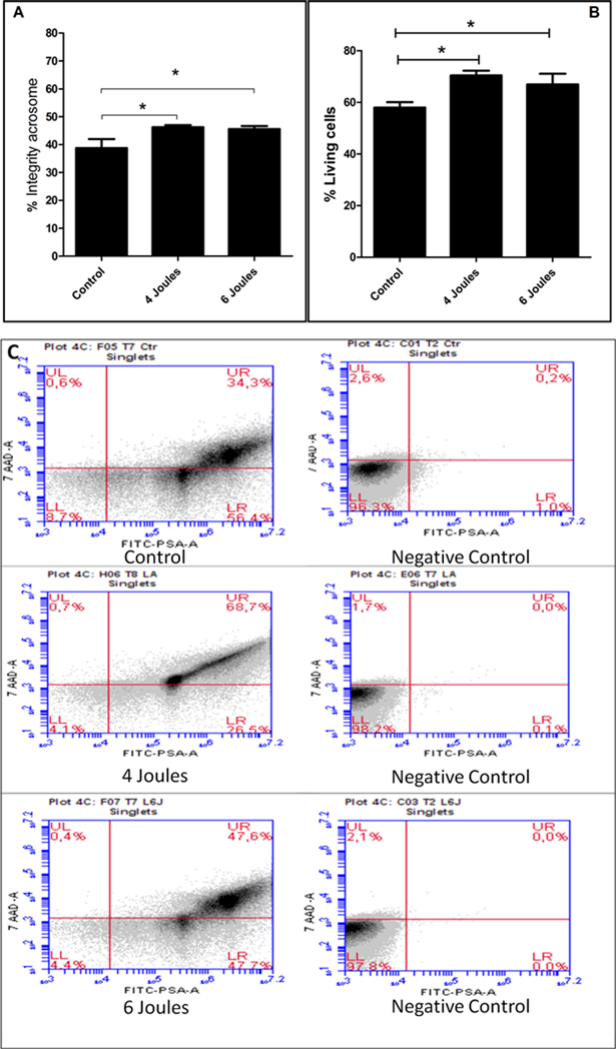
Representative Low Level Laser Irradiation in joules 4 and 6 and analyzed by flow cytometry. (A) Shows the percentage of sperm with acrosome integrity under the total of living cells. (Newman-Keuls Test * p < 0.05). (B) Shows the percentage of live sperm cells relative to the total cells analyzed. (C) Shows representative plots of the respective conditions when exposed to 7AAD and FITC-PSA. The quadrants indicate: UL—sperm dead (plasmatic membrane injured (MP) and acrosome membrane integrate (MA), RH—live sperm (plasmatic membrane injured (MP) and acrosome membrane integrate); LL—live (plasmatic membrane integrate (MP) and Membrane damaged acrosome (MA); LR—(plasmatic membrane injured (MP) and acrosome membrane integrate (MA), in the control, 4 joule and 6 joules groups.

Irradiation with a low-power laser significantly increased (*p* < 0.05) the percentage of live sperm cells as evaluated by flow cytometry, both in the 4 joule group (70.3 ± 4.8) and in the 6 joule group (66.9 ± 11.7) compared to the control group (57.9 ± 5.4), as shown in Fig. 1A). Similarly, low-power laser irradiation at both 4 joules (46.2 ± 2.2) and 6 joules (45.5 ± 3.3) maintained the integrity of the acrosome membrane of living cells, as assessed by flow cytometry, significantly better (*p* <0.05) when compared to the results obtained in the control group (38.7 ± 9.8) (Fig. 1B).

### Evaluation of sperm motility

**Fig 2:**
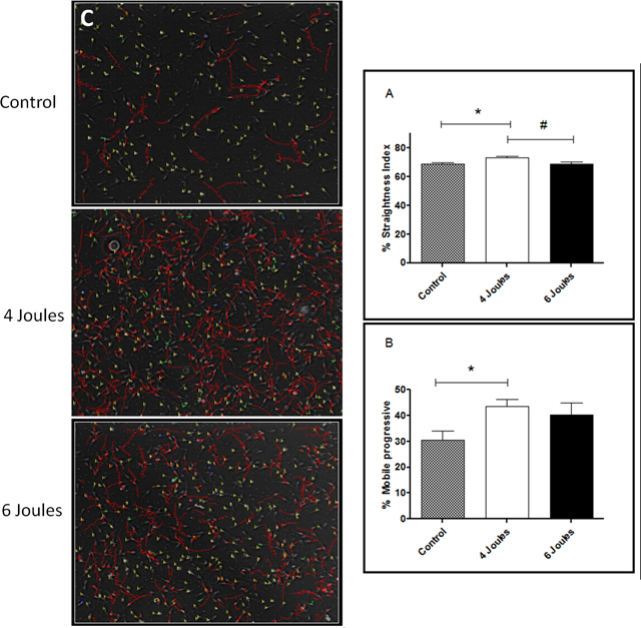
Evaluation of sperm movement through the Integrated Semen Analysis System in (A) Straightness Index percentage of sperm analysis presenting the non-irradiated control group and groups subjected to irradiation with low-power laser with dose 4:06 joules. Data shown are the mean ± standard deviation. One-way ANOVA and Newman-Keuls post hoc analysis were applied. * p < 0.05 control group vs. 4 Joules group and # p < 0.05. group 6 Joules vs. 4 Joules group. (B) Mobile percentage of progressive sperm analysis presenting the group not irradiated groups and subjected to low-level laser irradiation dose 4 and 6 joules group. Data shown are the mean ± standard deviation. One-way ANOVA and Newman-Keuls post hoc analysis were applied. * p <0.05 control group vs. 4 Joules group. (C) Graphical representation of sperm movement by Integrated Semen Analysis System, The red lines show the movements of the sperm in nm/s and the yellow lines represent the static sperm.

In comparing laser irradiation at doses of 4 and 6 joules, and a non-irradiated control group, the following variables were analyzed: curvilinear speed, rectilinear speed, average value, linearity index, oscillation index, head side movement, beat frequency, mobile progressive, and straightness index. On the straightness index variable, there was a statistical difference between the control group and the group irradiated with 4 joules and also between the two treatment groups, 4 and 6 joules (*p* <0.05). We also found statistical differences for the variable mobile progressive when comparing the control group (30.4 ± 10.5) with the 4 joules group (43.5 ± 7.7). The straightness index result also showed statistical significance (*p* < 0.05) between the control group (68.7 ± 3.7) and the 4 joules group (73.1 ± 3.0), and between the 4 joules group (73.1 ± 3.0) and the 6 joules group (68.7 ± 3.8) with *p* < 0.05 (Fig. 2). The results of the other outcomes related to motion analysis are summarized in Table 1.

**Table 1:** Results of the evaluation of sperm movement by the Integrated Semen Analysis System.

	Control	4J	6J
Curvilinear Speed (μ / s)	81±20	80±20	77±21
Rectilinear Speed (μ / s)	35±6	36±7	32±8
Average Value (μ / s)	50±10	50±10	47±12
Linearity Index %	42±3	46±5	41±6
Oscillation Index %	61±3	63±5	60±4
Head lateral moving (μ)	2.9±0.7	2.8±0.7	2.8±0.7
Beat Frequency (Hz)	10±1	10±1	9±2

Values are expressed as mean and standard deviation.

## Discussion

The rationale for this study addresses the difficulties faced by farmers in improving sperm viability and the possibility of low-power laser irradiation as a solution to this problem. Studies of the use of lasers in improving sperm viability, however, have presented conflicting results. Our goal was to evaluate the effects of two different doses of irradiation by low-power laser on sperm motility and on the integrity of the plasma membrane and acrosome in cryopreserved bovine sperm. Our results point to an increase in the percentage of live sperm cells and acrosome integrity in relation to control cells when subjected to irradiation by low-power laser in two different doses (4 and 6 joules). The analysis of straightness and the percentage of cell motility show that a dose of 4 joules is more effective.

In vitro production (IVP) of bovine embryos using frozen/thawed semen is used around the world for commercial purposes. Sperm cells are exposed to a series of potential risks during cryopreservation [21]. The freeze-thaw process damages the plasma membrane and the acrosome of the sperm [22]. One of the reasons proposed to explain this variation is a change in the integrity of the sperm chromatin [21]. Cryopreservation also leads to a reduction in size of the head of the sperm as compared to fresh semen, perhaps because of damage or loss of the acrosome or overcondensation of the nuclear chromatin of sperm [6].

Cryopreservation also significantly increases the production of reactive oxygen species (ROS) in sperm. ROS have two effects on sperm function: at low concentrations they induce sperm capacitation, hyperactivation of the acrosome, and sperm-oocyte fusion and, on the other hand, excessive amounts of ROS damage DNA, inhibit sperm-oocyte fusion, and reduce sperm motility [23].

Several studies have shown that LLLI accelerates wound healing [24], enhances repair of bone defects [25], modulates the production of inflammatory mediators in joint inflammation [26], and decreases oxidative stress and muscle fatigue [27]. Others have shown the LLLI improves the activation of anti-inflammatory vasoactive peptides [15] and increases cell energy and viability [13]. The mechanism of photobiostimulation by LLLI is still unclear. It has been suggested that reactive oxygen species (ROS), which can be produced by photosensitization of endogenous chromophores such as cell cytochromes, flavins/riboflavins, and NADPH, may have an important role in this light/tissue interaction [23]. Additionally, Albuquerque-Pontes *et al*. [27] demonstrated that cytochrome c oxidase (complex IV of mitochondrial respiratory chain) is modulated by different wavelengths and doses of LLLT at different time-intervals.

We previously noted that LLLI with the wavelength of 660 nm, power 30 mW and doses of 4 and 6 joules was able to improve the percentage of live sperm cells evaluated by flow cytometry and maintain acrosomal membrane integrity. A dose of 4 joules also increased the percentage of mobile progressive sperm and the straightness index.

The improvement in semen quality after LLLI Has Been illustrated previously described in several species: dog [16], bovine [28], [29], rabbit [30], and turkey. [31] Using the same energy and wavelength as in previous similar studies, we show additional evidence that LLLI may result in a significant increase in the percentage of live sperm cells, integrity of acrosome membrane, and higher sperm motility.

According to Karu *et al*. [13], the possible primary mechanisms of light activation of spermatozoids suggest that photoacceptors are connected with oxygen metabolism and, in particular, with respiratory chains. It is important to recall that respiratory chain molecules in eukaryotic as well as prokaryotic cells are considered photoacceptors and photosignal transducers in these cells.[13]. On the other hand Lubarte et al. [32] report that LLLI inhibits calcium uptake by mitochondria and stimulates calcium to connect the vesicles of the plasma membrane of the sperm, promoting better cell maintenance.

The analysis of the percentage of live sperm cells and the acrosome membrane integrity have been used in several other studies because they are important for the diagnosis of the viability of semen after cryopreservation [7], [9], [10], [22], [33]. However, few studies with LLLI [16], [30], [31] have used these factors to analyze the improved quality of semen. It is noteworthy that these studies also differ from our study in the form of measurements used, since we have fluorescein isothiocyanato-labeled *Pisum sativum* agglutinin (FITC-PSA) to detect the acrosome integrity by flow cytometry. Sperm motility after LLLI has been investigated in several studies [16], [30], [31], [33], [34]. However, some studies [30], [35] showed negative outcomes regarding motility, these results may be related to the wavelength, laser power, energy density, irradiation time, as well as the experimental analysis conditions. Considering the data presented in our study and the current state of knowledge regarding the efficacy of LLLI in improving the quality of semen, we conclude that LLLI may exert beneficial effects on both the preservation of live sperm and sperm motility after cryopreservation.

## Perspectives and Limitations

The low-level laser is used medically to accelerate repair processes of various types of tissue as well as to treat pain and inflammation. Pre-clinical studies have demonstrated several other possible uses. However, in vitro improvement in the quality of semen for artificial insemination has not been translated into actual practice. Possible mechanisms of low-level laser effects on the oxidative stress generated by cryopreservation include the following: (i) Superoxide anions induce hyperactivation and capacitation and are being altered by LLLI; (ii) capacitating spermatozoa produce elevated concentrations of superoxide anions themselves; and (iii) if the LLLI is capable of superoxide dismutase by removal of this ROS.
